# α-SrZn_5_-Type solid solution, BaZn_2.6_Cu_2.4_

**DOI:** 10.1107/S2056989019012532

**Published:** 2019-09-20

**Authors:** Rayko Simura, Hisanori Yamane

**Affiliations:** a Institute of Multidisciplinary Research for Advanced Materials, Tohoku University, 2-1-1 Katahira, Aoba-ku, Sendai, 980-8577, Japan

**Keywords:** crystal structure, barium zinc copper

## Abstract

Single crystals of the title compound barium zinc copper, BaCu_2.6_Zn_2.4_, were obtained from a sample prepared by heating metal chips of Ba, Cu, and Zn in an Ar atmosphere up to 973 K, followed by slow cooling. Single-crystal X-ray structure analysis revealed that BaCu_2.6_Zn_2.4_ crystallizes in an ortho­rhom­bic cell [*a* = 12.9858 (3), *b* = 5.2162 (1), and *c* = 6.6804 (2) Å] with an α-SrZn_5_-type structure (space group *Pnma*). The three-dimensional framework consists of Cu and Zn atoms, with Ba atoms in the tunnels extending in the *b*-axis direction. Although the Ba atom is larger than the Sr atom, the cell volume of BaCu_2.6_Zn_2.4_ [452.507 (19) Å^3^] is smaller than that of α-SrZn_5_ [466.08 Å^3^]. This decrease in volume can be attributed to the partial substitution of Cu atoms by Zn atoms in the framework because the Cu—Zn and Cu—Cu bonds are shorter than the Zn—Zn bond. The increase in Ba—Zn inter­atomic distances from the Sr—Zn distances is cancelled out by the partial replacement of Zn with Cu atoms, which leads to shorter average Ba—Zn/Cu distances.

## Abstract 2

BaCu_2.6_Zn_2.4_ has an α-SrZn_5_-type structure. Although the Ba atom is larger than the Sr atom, the cell volume of title compound is smaller than that of α-SrZn_5_. This can be attributed to the partial substitution of Cu atoms with Zn atoms, and the average Ba—Zn/Cu distance becomes shorter than the Sr—Zn distance.

## Chemical context   

In *A*–*M* binary systems (*A* = Ca, Sr, Ba, *M* = Zn, Cu), several phases are present such as *AM*, *AM*_5_, *AM*_11_, and *AM*_13_. *AM*_5_ phases appear except for *A* = Ba with *M* = Cu. CaZn_5_ (Häucke, 1940 ▸), CaCu_5_ (Häucke, 1940 ▸), β-SrZn_5_ (Bruzzone & Merlo, 1983 ▸), and SrCu_5_ (Bruzzone, 1966 ▸, 1971 ▸) crystallize in the hexa­gonal space group *P*6/*mmm*, and were reported to have the Kagome structure consisting of Zn or Cu atoms (Wendorff & Röhr, 2007 ▸). In addition to the high-temperature β-SrZn_5_ phase, there exists a low-temperature polymorph of α-SrZn_5_ in the ortho­rhom­bic space group *Pnma* (Baenziger & Conant, 1956 ▸; Bruzzone & Merlo, 1983 ▸; Wendorff & Röhr, 2007 ▸). BaZn_5_ is in the tetra­gonal space group *Cmcm* with a structure distorted from *P*6/*mmm*-type *AM*_5_ (Baenziger and Conant, 1956 ▸). In the present study, single crystals of a new ternary compound BaCu_2.6_Zn_2.4_ were synthesized, and the crystal structure was analyzed by X-ray diffraction.

## Structural commentary   

**Figure 1:**
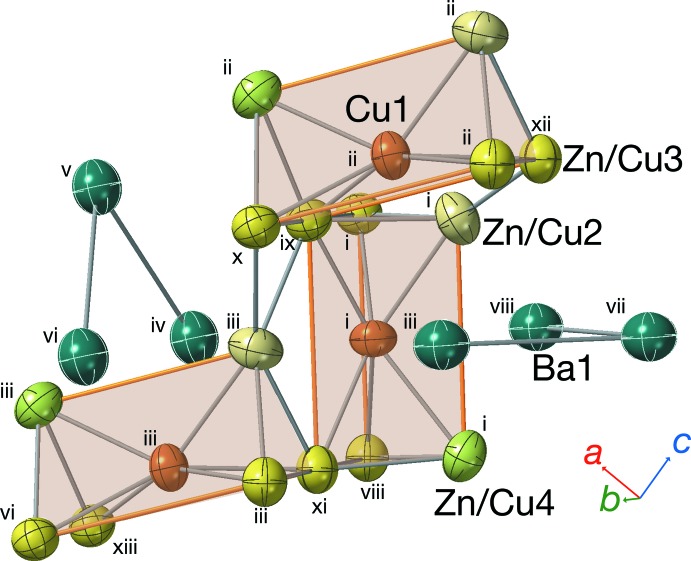
Arrangement of Cu1-centered Zn/Cu trigonal prisms and the Ba1 atoms. Symmetry codes: (i) *x*, *y*, *z*; (ii) 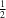 − *x*, 1 − *y*, 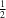 + *z*; (iii) 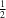 − *x*, 1 − *y*, −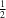 + *z*; (iv) *x*, *y*, −1 + *z*; (v) 1 − *x*, 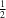 + *y*, 1 − *z*; (vi) *x*, 1 + *y*, −1 + *z*; (vii) −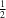 + *x*, 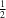 − *y*, 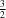 − *z*; (viii) 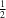 − *x*, −*y*, −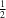 + *z*; (ix) *x*, 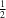 − *y*, *z*; (x) *x*, 1 + *y*, *z*; (xi) 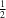 − *x*, 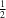 + *y*, −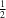 + *z*; (xii) 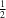 − *x*, 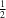 + *y*, 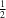 + *z*; (xiii) *x*, 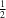 − *y*, −1 + *z*.

**Figure 2:**
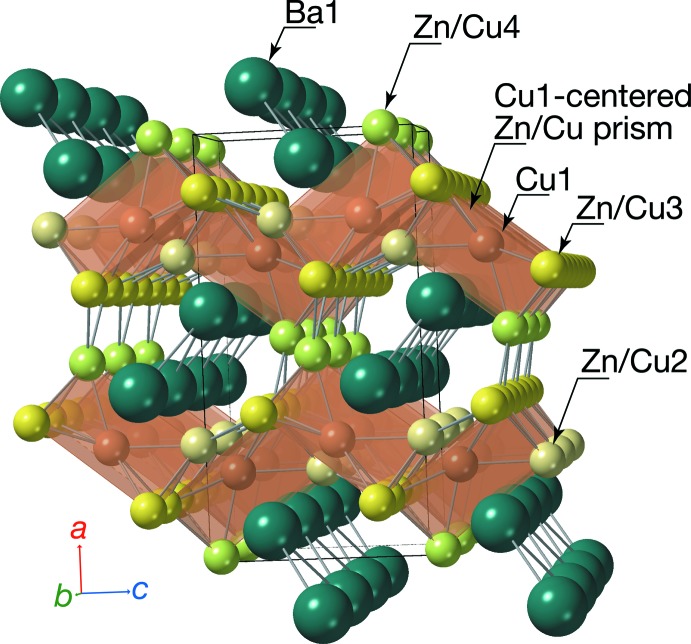
Crystal structure of BaCu_2.6_Zn_2.4_ illustrated with the Cu1-centered Zn/Cu trigonal prisms.

The volume for the chemical formula unit of the title compound (113.12 Å^3^ per formula) calculated from the cell volume *V* = 452.507 (19) Å^3^ and *Z* = 4 is smaller than that of BaZn_5_ (120.43 Å^3^ per formula). The asymmetric unit contains one Ba site (Ba1), one Cu site (Cu1), and three mixed sites of Zn and Cu (Zn/Cu2, Zn/Cu3, Zn/Cu4). As shown in Fig. 1 ▸, the Cu1 site is located inside the triangular prism composed of Zn/Cu2, Zn/Cu4, and four Zn/Cu3 sites. The refined occupancy for the Zn/Cu2 site is 0.735 (8)/0.265 (8), while the occupancies of Zn/Cu3 and Zn/Cu4 are almost equivalent at 0.555 (8)/0.445 (8) and 0.555 (4)/0.445 (4), respectively (Table 1 ▸). As shown in Table 2 ▸, the Cu1—Zn/Cu2 and Cu1—Zn/Cu4 bond lengths are 2.5958 (5) and 2.5840 (6) Å, respectively; Cu1—Zn/Cu3 bond lengths are 2.5664 (4) Å × 2 and 2.6001 (4) Å × 2. The average Cu—Zn/Cu distance is 2.5855 Å, which is shorter than the Zn1—Zn2, Zn1—Zn3, and Zn1—Zn4 distances in α-SrZn_5_ (2.6120 Å; Wendorff & Röhr 2007 ▸). The Zn/Cu2—Zn/Cu3 bond lengths are 2.5440 (4) Å × 2 and 2.5879 (4) Å × 2; Zn/Cu4—Zn/Cu3 bond lengths are 2.5576 (4) Å × 2 and 2.5756 (4) Å × 2; Zn/Cu3—Zn/Cu3 bond lengths are 2.6075 (5) and 2.6078 (5) Å; and the Zn/Cu4—Zn/Cu4 bond length is 2.8652 (3) Å × 2. These bonds are also shorter than those of SrZn_5_ [2.5622 (11) Å × 2 and 2.7260 (11) Å × 2; 2.5594 (11) Å × 2 and 2.6665 (11) Å × 2; 2.6452 (10) and 2.6539 (10) Å; 3.0018 (8) Å × 2], respectively. These shorter Cu—Zn/Cu bonds in BaCu_2.6_Zn_2.4_ are consistent with the Cu—Cu bond lengths in BaCu_13_ (2.49–2.68 Å, calculated using the data from Wendorff & Röhr, 2006 ▸), which are shorter than the Zn—Zn lengths for BaZn_13_ (2.60–2.94 Å, Bruzzone *et al.* 1985 ▸). The average Zn_/_Cu—Zn/Cu lengths for Ca(Zn_1-*x*_Cu_*x*_)_5_ (*x* = 0.97–0.6) decrease with increasing *x* (Merlo & Fornasini, 1985 ▸). In the title compound, the Cu1-centered triangular prisms align in the *b-* and *c*-axis directions by sharing the atoms of the Zn/Cu3 site, and form the framework of Cu and Zn atoms shown in Fig. 2 ▸.

The Ba1 sites are staggered along the array of the triangular prisms in the tunnel extending in the *b*-axis direction. The inter­atomic distance of Ba1—Ba1 [3.8503 (3) Å] is shorter than that of Sr1—Sr1 [4.0230 (13) Å] of α-SrZn_5._ The Ba1—Ba1—Ba1 angle [85.279 (9)°] is comparable to the Sr1—Sr1—Sr1 angle of α-SrZn_5_ [82.39 (3)°]. There are three Cu1 sites, three Zn/Cu2 sites, eight Zn/Cu3 sites, and three Zn/Cu4 sites around the Ba1 site (Table 2 ▸). The average distance of inter­atomic distances between these 17 sites and the Ba1 site (3.495 Å) is shorter than that between 17 Zn sites and the Ba site of BaZn_5_ (3.5832 Å), but is the same as that between 17 Sr sites and the Sr site of α-SrZn_5_ (3.495 Å).

Comparison between BaCu_13_ and BaZn_13_ having an isotypic structure showed that the atomic distance of Ba—Cu (3.42 Å, calculated using the data by Wendorff & Röhr, 2006 ▸) is shorter than that of Ba—Zn (3.59 Å, calculated using the data by Bruzzone *et al.*, 1985 ▸). This result is consistent with the fact that the average distance between the Ba1 site and Cu or Zn/Cu sites becomes shorter than the average atomic distance of Ba—Zn in BaZn_5_. The lattice size of BaCu_2.6_Zn_2.4_ is expected to be larger than that of α-SrZn_5_ because the inter­atomic distance of Ba—Zn for BaZn_13_ is longer than that of Sr—Zn for SrZn_13_, both of which have the same type of structure. However, the cell constants and volume for BaCu_2.6_Zn_2.4_ are 0.3–2.0% and 3.0–3.9% smaller than those reported for α-SrZn_5_ [*a* = 13.15 (4), *b* = 5.32 (1), *c* = 6.72 (2) Å, *V* = 470.12 Å^3^ (Baenziger & Conant, 1956 ▸); *a* = 13.147 (7), *b* = 5.312 (2), *c* = 6.707 (3) Å, *V* = 468.4 Å^3^ (Bruzzone & Merlo, 1983 ▸); and *a* = 13.133 (3), *b* = 5.2991 (10), *c* = 6.6972 (13) Å, *V* = 466.08 Å^3^ (Wendorff & Röhr, 2007 ▸)]. The average inter­atomic distance between Ba and the framework forming Zn/Cu atoms in the title compound remains the same as the average Sr—Zn distance of α-SrZn_5_ by partial substitution of Cu with Zn atoms. Thus, the decrease in cell volume is caused by the introduction of the shorter Cu—Zn and Cu—Cu bonds in the BaCu_2.6_Zn_2.4_ framework.

## Synthesis and crystallization   

The title compound was prepared from pieces of Ba (Aldrich Chemicals, 99.9%), Cu (Kojundo Chemical Laboratory Co., Ltd., 99.99%), and Zn (Strem Chemicals Inc., 99.99%) metals with molar ratio of Ba:Cu:Zn = 1:1:1. The metals were placed in a BN crucible (Showa Denko Co., Ltd., purity 99.95%, outer diameter 8.5 mm, inner diameter 6.5 mm, depth 18 mm), which was then put inside a stainless-steel tube (SUS 316: outer diameter 12.7 mm, inner diameter 10.7 mm, height 80 mm) and sealed with a stainless-steel cap in an Ar-filled glove box (MBRAUN; O_2_ and H_2_O < 1 ppm). The tube was heated to 933 K at a rate of 330 Kh^−1^ for 10 h, then slowly cooled at a rate of 10 Kh^−1^ to below 573 K. Finally, the sample was cooled to room temperature by shutting off the electric power to the heater of the furnace. The stainless-steel tube was cut in the Ar-filled glove box. The resulting product contained silver metallic single crystals of the title compound with size of several hundred µm. The surface color of the single crystals changed to metallic gold in air, but crystal decomposition did not occur. A thin layer formed by oxidation may have prevented the further oxidation of the sample. Single crystal XRD data collection was carried out in air. Another single crystal grain obtained from the same sample was buried in resin and polished with a SiC polishing sheet to verify the composition of the crystal by electron probe microanalysis (EPMA, JEOL JXA-8200). A Ba:Cu:Zn atomic ratio of 1.01 ± 0.01:2.62 ± 0.10:2.37 ± 0.09 was obtained by measurements at seven points with the total weight percent of 94–98%. From the EPMA measurement, the chemical composition of the single crystal was determined to be BaCu_2.6_Zn_2.4_.

## Refinement   

Crystal data, data collection, and structural refinement details are summarized in Table 3 ▸. The initial structural model was constructed from the α-SrZn_5_ model by substituting the Ba1 site with the Sr1 site, and the Zn/Cu mixed sites with the four Zn sites. In the first stage of refinement, the sum of the occupancies for Cu and Zn atoms in each Zn/Cu site was constrained to be 1. After several refinement iterations, the Cu occupancy for the Zn/Cu1 site became 0.98 (6), and then this site was set to be fully occupied by Cu only. The substitutional occupations of the other three Zn/Cu mixed sites were refined under the restriction that the total chemical composition should be Zn:Cu = 0.48:0.52, which was determined by EPMA.

## Supplementary Material

Crystal structure: contains datablock(s) I. DOI: 10.1107/S2056989019012532/vn2152sup1.cif

Structure factors: contains datablock(s) I. DOI: 10.1107/S2056989019012532/vn2152Isup2.hkl

CCDC reference: 1952238

Additional supporting information:  crystallographic information; 3D view; checkCIF report

## Figures and Tables

**Table 1:** Fractional atomic coordinates and equivalent isotropic displacement parameters for BaCu_2.6_Zn_2.4_

Atom	*x*	*y*	*z*	*U_eq_*	Occupancy
Ba1	0.41339 (2)	1/4	0.87119 (3)	0.01724 (6)	1
Cu1	0.21263 (3)	1/4	0.17198 (6)	0.01346 (8)	1
Zn/Cu2	0.21485 (3)	1/4	0.56053 (6)	0.01585 (9)	0.555 (8)/0.445 (8)
Zn/Cu3	0.35265 (2)	−0.00006 (5)	0.36005 (4)	0.01279 (7)	0.555 (4)/0.445 (4)
Zn/Cu4	0.01929 (3)	1/4	0.08049 (6)	0.01551 (9)	0.735 (8)/0.265 (8)

**Table 2:** Selected geometric parameters (Å) for BaCu_2.6_Zn_2.4_

Ba1—Cu1^i^	3.2916 (5)	Cu1—Zn/Cu3^ix^	2.5664 (4)
Ba1—Zn/Cu2	3.3097 (5)	Cu1—Zn/Cu3	2.5664 (4)
Ba1—Zn/Cu4^ii^	3.3160 (5)	Cu1—Zn/Cu4	2.5840 (6)
Ba1—Zn/Cu2^iii^	3.3429 (3)	Cu1—Zn/Cu2	2.5958 (5)
Ba1—Zn/Cu2^iv^	3.3429 (3)	Cu1—Zn/Cu3^xii^	2.6001 (4)
Ba1—Cu1^iii^	3.3542 (3)	Cu1—Zn/Cu3^xiii^	2.6001 (4)
Ba1—Cu1^iv^	3.3542 (3)	Zn/Cu2—Zn/Cu3^viii^	2.5440 (4)
Ba1—Zn/Cu4^iii^	3.3672 (3)	Zn/Cu2—Zn/Cu3^iii^	2.5440 (4)
Ba1—Zn/Cu4^iv^	3.3672 (3)	Zn/Cu2—Zn/Cu3	2.5879 (4)
Ba1—Zn/Cu3^v^	3.6040 (3)	Zn/Cu2—Zn/Cu3^ix^	2.5879 (4)
Ba1—Zn/Cu3^i^	3.6040 (3)	Zn/Cu3—Zn/Cu3^xiv^	2.6075 (5)
Ba1—Zn/Cu3^vi^	3.6492 (3)	Zn/Cu3—Zn/Cu3^ix^	2.6087 (5)
Ba1—Zn/Cu3^vii^	3.6492 (3)	Zn/Cu4—Zn/Cu3^xv^	2.5576 (4)
Ba1—Zn/Cu3^iii^	3.6933 (3)	Zn/Cu4—Zn/Cu3^xvi^	2.5576 (4)
Ba1—Zn/Cu3^viii^	3.6933 (3)	Zn/Cu4—Zn/Cu3^xii^	2.5756 (4)
Ba1—Zn/Cu3	3.7394 (3)	Zn/Cu4—Zn/Cu3^xiii^	2.5756 (4)
Ba1—Zn/Cu3^ix^	3.7394 (3)	Zn/Cu4—Zn/Cu4^xvii^	2.8652 (3)
Ba1—Ba1^*x*^	3.8503 (3)	Zn/Cu4—Zn/Cu4^xviii^	2.8652 (3)
Ba1—Ba1^xi^	3.8502 (3)		
			
Ba1^*x*^—Ba1—Ba1^xi^	85.279 (9)		

Symmetry codes: (i) *x*, *y*, *z* + 1; (ii) *x* + 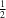, *y*, −*z* + 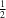; (iii) −*x* + 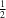, −*y*, *z* + 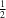; (iv) −*x* + 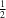, −*y* + 1, *z* + 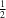; (v) *x*, −*y* + 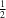, *z* + 1; (vi) −*x* + 1, *y* + 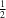, −*z* + 1; (vii) −*x* + 1, −*y*, −*z* + 1; (viii) −*x* + 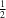, *y* + 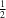, *z* + 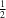; (ix) *x*, −*y* + 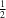, *z*; (*x*) −*x* + 1, −*y* + 1, −*z* + 2; (xi) −*x* + 1, −*y*, −*z* + 2; (xii) −*x* + 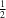, −*y*, *z* − 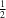; (xiii) −*x* + 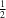, *y* + 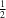, *z* − 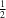; (xiv) *x*, −*y* − 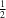, *z*; (xv) *x* − 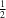, *y*, −*z* + 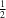; (xvi) *x* − 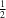, −*y* + 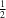, −*z* + 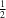; (xvii) −*x*, −*y*, −*z*; (xviii) −*x*, −*y* + 1, −*z*.

**Table 3:** Experimental details

Crystal data	
Chemical formula	BaCu_2.60_Zn_2.40_
*M*_r_	459.5
Crystal system, space group	Orthorhombic, *P* *n* *m* *a*
Temperature (K)	300
*a*, *b*, *c* (Å)	12.9858 (3), 5.2162 (1), 6.6804 (2)
*V* (Å^3^)	452.51 (2)
*Z*	4
Radiation type	Mo *K*α
μ (mm^−1^)	32.87
Crystal size (mm)	0.10 × 0.07 × 0.06

Data collection	
Diffractometer	Bruker D8 QUEST
Absorption correction	Multi-scan (*SADABS*; Bruker, 1997 ▸)
*T*_min_, *T*_max_	0.49, 0.75
No. of measured, independent and observed [*I* > 2σ(*I*)] reflections	9003, 1038, 956
*R*_int_	0.029
(sin θ/λ)_max_ (Å^−1^)	0.794

Refinement	
*R*[*F* ^2^ > 2σ(*F* ^2^)], *wR*(*F* ^2^), *S*	0.017, 0.032, 1.17
No. of reflections	1038
No. of parameters	39
No. of restraints	1
Δρ_max_, Δρ_min_ (e Å^−3^)	0.96, −0.98

Computer programs: *APEX3* (Bruker, 2018 ▸), *SAINT* (Bruker, 1997 ▸), *SHELXT2014/5* (Sheldrick, 2015*a* ▸), *SHELXL2014/7* (Sheldrick, 2015*b* ▸), *Crystal Maker X* (CrystalMaker Software, 2003 ▸), *publCIF* (Westrip, 2010 ▸).

## supplementary crystallographic information

### Crystal data

**Table:**

BaCu_2.60_Zn_2.40_	*D*_x_ = 6.744 Mg m^−^^3^
*M**_r_* = 459.5	Mo *K*α radiation, λ = 0.71073 Å
Orthorhombic, *P**n**m**a*	Cell parameters from 198 reflections
*a* = 12.9858 (3) Å	θ = 3.3–27.3°
*b* = 5.2162 (1) Å	µ = 32.87 mm^−^^1^
*c* = 6.6804 (2) Å	*T* = 300 K
*V* = 452.51 (2) Å^3^	Chip, metallic light silver
*Z* = 4	0.10 × 0.07 × 0.06 mm
*F*(000) = 813.6	

### Data collection

**Table:**

Bruker D8 QUEST diffractometer	956 reflections with *I* > 2σ(*I*)
Detector resolution: 7.3910 pixels mm^-1^	*R*_int_ = 0.029
ω and σcans	θ_max_ = 34.4°, θ_min_ = 3.1°
Absorption correction: multi-scan (*SADABS*; Bruker, 1997)	*h* = −20→20
*T*_min_ = 0.49, *T*_max_ = 0.75	*k* = −8→7
9003 measured reflections	*l* = −10→10
1038 independent reflections	

### Refinement

**Table:**

Refinement on *F*^2^	1 restraint
Least-squares matrix: full	*w* = 1/[σ^2^(*F*_o_^2^) + (0.0069*P*)^2^ + 0.6738*P*] where *P* = (*F*_o_^2^ + 2*F*_c_^2^)/3
*R*[*F*^2^ > 2σ(*F*^2^)] = 0.017	(Δ/σ)_max_ = 0.003
*wR*(*F*^2^) = 0.032	Δρ_max_ = 0.96 e Å^−^^3^
*S* = 1.17	Δρ_min_ = −0.98 e Å^−^^3^
1038 reflections	Extinction correction: SHELXL-2014/7 (Sheldrick 2015b, Fc^*^=kFc[1+0.001xFc^2^λ^3^/sin(2θ)]^-1/4^
39 parameters	Extinction coefficient: 0.00318 (18)

### Special details

**Table:**

Geometry. All esds (except the esd in the dihedral angle between two l.s. planes) are estimated using the full covariance matrix. The cell esds are taken into account individually in the estimation of esds in distances, angles and torsion angles; correlations between esds in cell parameters are only used when they are defined by crystal symmetry. An approximate (isotropic) treatment of cell esds is used for estimating esds involving l.s. planes.

### Fractional atomic coordinates and isotropic or equivalent isotropic displacement parameters (Å^2^)

**Table:**

	*x*	*y*	*z*	*U*_iso_*/*U*_eq_	Occ. (<1)
Ba1	0.41339 (2)	0.2500	0.87119 (3)	0.01724 (6)	
Cu1	0.21263 (3)	0.2500	0.17198 (6)	0.01346 (8)	
Zn2	0.21485 (3)	0.2500	0.56053 (6)	0.01585 (9)	0.555 (8)
Cu2	0.21485 (3)	0.2500	0.56053 (6)	0.01585 (9)	0.445 (8)
Zn3	0.35265 (2)	−0.00006 (5)	0.36005 (4)	0.01279 (7)	0.555 (4)
Cu3	0.35265 (2)	−0.00006 (5)	0.36005 (4)	0.01279 (7)	0.445 (4)
Zn4	0.01929 (3)	0.2500	0.08049 (6)	0.01551 (9)	0.735 (8)
Cu4	0.01929 (3)	0.2500	0.08049 (6)	0.01551 (9)	0.265 (8)

### Atomic displacement parameters (Å^2^)

**Table:**

	*U*^11^	*U*^22^	*U*^33^	*U*^12^	*U*^13^	*U*^23^
Ba1	0.01853 (10)	0.01541 (10)	0.01779 (10)	0.000	0.00248 (7)	0.000
Cu1	0.01259 (17)	0.01619 (19)	0.01161 (16)	0.000	−0.00309 (13)	0.000
Zn2	0.01913 (19)	0.01558 (19)	0.01283 (17)	0.000	0.00354 (14)	0.000
Cu2	0.01913 (19)	0.01558 (19)	0.01283 (17)	0.000	0.00354 (14)	0.000
Zn3	0.01308 (12)	0.01109 (13)	0.01420 (13)	0.00054 (9)	−0.00154 (9)	0.00036 (9)
Cu3	0.01308 (12)	0.01109 (13)	0.01420 (13)	0.00054 (9)	−0.00154 (9)	0.00036 (9)
Zn4	0.01137 (16)	0.01599 (19)	0.01916 (19)	0.000	0.00157 (13)	0.000
Cu4	0.01137 (16)	0.01599 (19)	0.01916 (19)	0.000	0.00157 (13)	0.000

### Geometric parameters (Å, º)

**Table:**

Ba1—Cu1^i^	3.2916 (5)	Cu1—Zn3^vii^	2.6001 (4)
Ba1—Zn2	3.3097 (5)	Cu1—Zn3^viii^	2.6001 (4)
Ba1—Zn4^ii^	3.3160 (5)	Cu1—Zn2^ix^	2.8711 (2)
Ba1—Zn2^iii^	3.3429 (3)	Cu1—Zn2^vii^	2.8711 (3)
Ba1—Zn2^iv^	3.3429 (3)	Zn2—Zn3^x^	2.5440 (4)
Ba1—Cu1^iii^	3.3542 (3)	Zn2—Zn3^iii^	2.5440 (4)
Ba1—Cu1^iv^	3.3542 (3)	Zn2—Zn3	2.5879 (4)
Ba1—Zn4^iii^	3.3672 (3)	Zn2—Zn3^vi^	2.5879 (4)
Ba1—Zn4^iv^	3.3672 (3)	Zn3—Zn4^ii^	2.5576 (4)
Ba1—Zn3^v^	3.6040 (3)	Zn3—Zn4^iii^	2.5756 (4)
Ba1—Zn3^i^	3.6040 (3)	Zn3—Zn3^xi^	2.6075 (5)
Cu1—Zn3^vi^	2.5664 (4)	Zn3—Zn3^vi^	2.6087 (5)
Cu1—Zn3	2.5664 (4)	Zn4—Zn4^xii^	2.8652 (3)
Cu1—Zn4	2.5840 (6)	Zn4—Zn4^xiii^	2.8652 (3)
Cu1—Zn2	2.5958 (5)		
			
Cu1^i^—Ba1—Zn2	76.456 (11)	Zn3^iii^—Zn2—Ba1^ix^	121.722 (15)
Cu1^i^—Ba1—Zn4^ii^	152.125 (12)	Zn3—Zn2—Ba1^ix^	122.810 (14)
Zn2—Ba1—Zn4^ii^	75.669 (11)	Zn3^vi^—Zn2—Ba1^ix^	75.843 (9)
Cu1^i^—Ba1—Zn2^iii^	51.279 (6)	Cu1—Zn2—Ba1^ix^	67.425 (9)
Zn2—Ba1—Zn2^iii^	81.327 (5)	Cu1^iv^—Zn2—Ba1^ix^	63.436 (8)
Zn4^ii^—Ba1—Zn2^iii^	123.428 (7)	Cu1^iii^—Zn2—Ba1^ix^	165.984 (15)
Cu1^i^—Ba1—Zn2^iv^	51.279 (6)	Ba1—Zn2—Ba1^ix^	128.704 (6)
Zn2—Ba1—Zn2^iv^	81.327 (5)	Ba1^vii^—Zn2—Ba1^ix^	102.556 (13)
Zn4^ii^—Ba1—Zn2^iv^	123.428 (7)	Zn3^x^—Zn2—Ba1^xv^	64.323 (11)
Zn2^iii^—Ba1—Zn2^iv^	102.555 (13)	Zn3^iii^—Zn2—Ba1^xv^	64.323 (11)
Cu1^i^—Ba1—Cu1^iii^	81.710 (5)	Zn3—Zn2—Ba1^xv^	138.309 (12)
Zn2—Ba1—Cu1^iii^	51.038 (6)	Zn3^vi^—Zn2—Ba1^xv^	138.309 (12)
Zn4^ii^—Ba1—Cu1^iii^	80.868 (9)	Cu1—Zn2—Ba1^xv^	96.009 (15)
Zn2^iii^—Ba1—Cu1^iii^	45.610 (9)	Cu1^iv^—Zn2—Ba1^xv^	107.206 (11)
Zn2^iv^—Ba1—Cu1^iii^	120.913 (11)	Cu1^iii^—Zn2—Ba1^xv^	107.206 (11)
Cu1^i^—Ba1—Cu1^iv^	81.710 (5)	Ba1—Zn2—Ba1^xv^	134.520 (11)
Zn2—Ba1—Cu1^iv^	51.038 (6)	Ba1^vii^—Zn2—Ba1^xv^	63.193 (8)
Zn4^ii^—Ba1—Cu1^iv^	80.868 (9)	Ba1^ix^—Zn2—Ba1^xv^	63.193 (8)
Zn2^iii^—Ba1—Cu1^iv^	120.913 (11)	Zn2^vii^—Zn3—Zn4^ii^	132.457 (17)
Zn2^iv^—Ba1—Cu1^iv^	45.610 (9)	Zn2^vii^—Zn3—Cu1	68.362 (11)
Cu1^iii^—Ba1—Cu1^iv^	102.074 (12)	Zn4^ii^—Zn3—Cu1	114.601 (13)
Cu1^i^—Ba1—Zn4^iii^	123.899 (7)	Zn2^vii^—Zn3—Zn4^iii^	114.379 (13)
Zn2—Ba1—Zn4^iii^	80.829 (9)	Zn4^ii^—Zn3—Zn4^iii^	67.858 (9)
Zn4^ii^—Ba1—Zn4^iii^	50.766 (6)	Cu1—Zn3—Zn4^iii^	174.066 (17)
Zn2^iii^—Ba1—Zn4^iii^	75.123 (8)	Zn2^vii^—Zn3—Zn2	115.282 (11)
Zn2^iv^—Ba1—Zn4^iii^	162.151 (12)	Zn4^ii^—Zn3—Zn2	104.336 (14)
Cu1^iii^—Ba1—Zn4^iii^	45.217 (9)	Cu1—Zn3—Zn2	60.479 (14)
Cu1^iv^—Ba1—Zn4^iii^	120.008 (10)	Zn4^iii^—Zn3—Zn2	113.935 (15)
Cu1^i^—Ba1—Zn4^iv^	123.899 (7)	Zn2^vii^—Zn3—Cu1^iii^	105.132 (14)
Zn2—Ba1—Zn4^iv^	80.829 (9)	Zn4^ii^—Zn3—Cu1^iii^	114.021 (16)
Zn4^ii^—Ba1—Zn4^iv^	50.766 (6)	Cu1—Zn3—Cu1^iii^	114.595 (11)
Zn2^iii^—Ba1—Zn4^iv^	162.151 (12)	Zn4^iii^—Zn3—Cu1^iii^	59.898 (13)
Zn2^iv^—Ba1—Zn4^iv^	75.123 (8)	Zn2—Zn3—Cu1^iii^	67.204 (10)
Cu1^iii^—Ba1—Zn4^iv^	120.008 (10)	Zn2^vii^—Zn3—Zn3^xi^	59.170 (7)
Cu1^iv^—Ba1—Zn4^iv^	45.217 (9)	Zn4^ii^—Zn3—Zn3^xi^	120.663 (8)
Zn4^iii^—Ba1—Zn4^iv^	101.532 (12)	Cu1—Zn3—Zn3^xi^	120.547 (7)
Cu1^i^—Ba1—Zn3^v^	43.406 (7)	Zn4^iii^—Zn3—Zn3^xi^	59.590 (7)
Zn2—Ba1—Zn3^v^	113.437 (9)	Zn2—Zn3—Zn3^xi^	120.266 (7)
Zn4^ii^—Ba1—Zn3^v^	156.251 (6)	Cu1^iii^—Zn3—Zn3^xi^	59.906 (7)
Zn2^iii^—Ba1—Zn3^v^	80.241 (8)	Zn2^vii^—Zn3—Zn3^vi^	120.829 (7)
Zn2^iv^—Ba1—Zn3^v^	42.758 (8)	Zn4^ii^—Zn3—Zn3^vi^	59.337 (8)
Cu1^iii^—Ba1—Zn3^v^	122.288 (9)	Cu1—Zn3—Zn3^vi^	59.453 (7)
Cu1^iv^—Ba1—Zn3^v^	88.359 (8)	Zn4^iii^—Zn3—Zn3^vi^	120.410 (7)
Zn4^iii^—Ba1—Zn3^v^	149.290 (8)	Zn2—Zn3—Zn3^vi^	59.734 (7)
Zn4^iv^—Ba1—Zn3^v^	107.404 (7)	Cu1^iii^—Zn3—Zn3^vi^	120.095 (7)
Cu1^i^—Ba1—Zn3^i^	43.406 (7)	Zn3^xi^—Zn3—Zn3^vi^	180.0
Zn2—Ba1—Zn3^i^	113.437 (10)	Zn2^vii^—Zn3—Ba1^xiv^	63.138 (9)
Zn4^ii^—Ba1—Zn3^i^	156.251 (6)	Zn4^ii^—Zn3—Ba1^xiv^	76.764 (11)
Zn2^iii^—Ba1—Zn3^i^	42.758 (8)	Cu1—Zn3—Ba1^xiv^	61.802 (11)
Zn2^iv^—Ba1—Zn3^i^	80.241 (8)	Zn4^iii^—Zn3—Ba1^xiv^	124.057 (13)
Cu1^iii^—Ba1—Zn3^i^	88.359 (8)	Zn2—Zn3—Ba1^xiv^	115.967 (11)
Cu1^iv^—Ba1—Zn3^i^	122.288 (9)	Cu1^iii^—Zn3—Ba1^xiv^	168.248 (12)
Zn4^iii^—Ba1—Zn3^i^	107.404 (7)	Zn3^xi^—Zn3—Ba1^xiv^	111.218 (4)
Zn4^iv^—Ba1—Zn3^i^	149.290 (8)	Zn3^vi^—Zn3—Ba1^xiv^	68.782 (4)
Zn3^v^—Ba1—Zn3^i^	42.436 (9)	Zn2^vii^—Zn3—Ba1^xvi^	76.752 (12)
Zn3^vi^—Cu1—Zn3	61.093 (15)	Zn4^ii^—Zn3—Ba1^xvi^	62.842 (9)
Zn3^vi^—Cu1—Zn4	143.536 (12)	Cu1—Zn3—Ba1^xvi^	124.350 (13)
Zn3—Cu1—Zn4	143.535 (12)	Zn4^iii^—Zn3—Ba1^xvi^	61.552 (11)
Zn3^vi^—Cu1—Zn2	60.171 (12)	Zn2—Zn3—Ba1^xvi^	167.121 (13)
Zn3—Cu1—Zn2	60.171 (12)	Cu1^iii^—Zn3—Ba1^xvi^	115.565 (11)
Zn4—Cu1—Zn2	104.319 (19)	Zn3^xi^—Zn3—Ba1^xvi^	69.067 (4)
Zn3^vi^—Cu1—Zn3^vii^	151.424 (19)	Zn3^vi^—Zn3—Ba1^xvi^	110.932 (4)
Zn3—Cu1—Zn3^vii^	111.623 (8)	Ba1^xiv^—Zn3—Ba1^xvi^	64.121 (6)
Zn4—Cu1—Zn3^vii^	59.580 (12)	Zn2^vii^—Zn3—Ba1^vii^	60.831 (11)
Zn2—Cu1—Zn3^vii^	143.610 (13)	Zn4^ii^—Zn3—Ba1^vii^	165.534 (13)
Zn3^vi^—Cu1—Zn3^viii^	111.623 (8)	Cu1—Zn3—Ba1^vii^	61.739 (9)
Zn3—Cu1—Zn3^viii^	151.425 (19)	Zn4^iii^—Zn3—Ba1^vii^	114.440 (11)
Zn4—Cu1—Zn3^viii^	59.580 (12)	Zn2—Zn3—Ba1^vii^	61.359 (9)
Zn2—Cu1—Zn3^viii^	143.610 (13)	Cu1^iii^—Zn3—Ba1^vii^	60.125 (11)
Zn3^vii^—Cu1—Zn3^viii^	60.188 (14)	Zn3^xi^—Zn3—Ba1^vii^	69.329 (4)
Zn3^vi^—Cu1—Zn2^ix^	55.449 (11)	Zn3^vi^—Zn3—Ba1^vii^	110.672 (4)
Zn3—Cu1—Zn2^ix^	110.868 (16)	Ba1^xiv^—Zn3—Ba1^vii^	110.530 (8)
Zn4—Cu1—Zn2^ix^	104.914 (12)	Ba1^xvi^—Zn3—Ba1^vii^	131.391 (8)
Zn2—Cu1—Zn2^ix^	104.812 (11)	Zn3^xvii^—Zn4—Zn3^xviii^	61.325 (15)
Zn3^vii^—Cu1—Zn2^ix^	110.769 (15)	Zn3^xvii^—Zn4—Zn3^viii^	153.278 (18)
Zn3^viii^—Cu1—Zn2^ix^	56.195 (11)	Zn3^xviii^—Zn4—Zn3^viii^	112.142 (9)
Zn3^vi^—Cu1—Zn2^vii^	110.868 (16)	Zn3^xvii^—Zn4—Zn3^vii^	112.142 (9)
Zn3—Cu1—Zn2^vii^	55.449 (11)	Zn3^xviii^—Zn4—Zn3^vii^	153.278 (18)
Zn4—Cu1—Zn2^vii^	104.914 (12)	Zn3^viii^—Zn4—Zn3^vii^	60.821 (15)
Zn2—Cu1—Zn2^vii^	104.812 (11)	Zn3^xvii^—Zn4—Cu1	141.749 (13)
Zn3^vii^—Cu1—Zn2^vii^	56.195 (11)	Zn3^xviii^—Zn4—Cu1	141.749 (13)
Zn3^viii^—Cu1—Zn2^vii^	110.769 (15)	Zn3^viii^—Zn4—Cu1	60.522 (12)
Zn2^ix^—Cu1—Zn2^vii^	130.57 (2)	Zn3^vii^—Zn4—Cu1	60.522 (12)
Zn3^vi^—Cu1—Ba1^xiv^	74.792 (12)	Zn3^xvii^—Zn4—Zn4^xii^	56.370 (13)
Zn3—Cu1—Ba1^xiv^	74.792 (12)	Zn3^xviii^—Zn4—Zn4^xii^	112.00 (2)
Zn4—Cu1—Ba1^xiv^	128.695 (16)	Zn3^viii^—Zn4—Zn4^xii^	111.04 (2)
Zn2—Cu1—Ba1^xiv^	126.987 (17)	Zn3^vii^—Zn4—Zn4^xii^	55.772 (13)
Zn3^vii^—Cu1—Ba1^xiv^	76.642 (12)	Cu1—Zn4—Zn4^xii^	104.989 (16)
Zn3^viii^—Cu1—Ba1^xiv^	76.642 (12)	Zn3^xvii^—Zn4—Zn4^xiii^	112.00 (2)
Zn2^ix^—Cu1—Ba1^xiv^	65.285 (11)	Zn3^xviii^—Zn4—Zn4^xiii^	56.370 (13)
Zn2^vii^—Cu1—Ba1^xiv^	65.285 (11)	Zn3^viii^—Zn4—Zn4^xiii^	55.772 (13)
Zn3^x^—Zn2—Zn3^iii^	61.660 (15)	Zn3^vii^—Zn4—Zn4^xiii^	111.04 (2)
Zn3^x^—Zn2—Zn3	154.64 (2)	Cu1—Zn4—Zn4^xiii^	104.989 (16)
Zn3^iii^—Zn2—Zn3	112.768 (8)	Zn4^xii^—Zn4—Zn4^xiii^	131.08 (3)
Zn3^x^—Zn2—Zn3^vi^	112.768 (8)	Zn3^xvii^—Zn4—Ba1^xvii^	77.904 (12)
Zn3^iii^—Zn2—Zn3^vi^	154.64 (2)	Zn3^xviii^—Zn4—Ba1^xvii^	77.904 (12)
Zn3—Zn2—Zn3^vi^	60.531 (15)	Zn3^viii^—Zn4—Ba1^xvii^	75.374 (12)
Zn3^x^—Zn2—Cu1	141.503 (14)	Zn3^vii^—Zn4—Ba1^xvii^	75.374 (12)
Zn3^iii^—Zn2—Cu1	141.503 (14)	Cu1—Zn4—Ba1^xvii^	128.184 (17)
Zn3—Zn2—Cu1	59.350 (12)	Zn4^xii^—Zn4—Ba1^xvii^	65.542 (15)
Zn3^vi^—Zn2—Cu1	59.350 (12)	Zn4^xiii^—Zn4—Ba1^xvii^	65.542 (15)
Zn3^x^—Zn2—Cu1^iv^	56.189 (10)	Zn3^xvii^—Zn4—Ba1^ix^	121.676 (15)
Zn3^iii^—Zn2—Cu1^iv^	112.000 (16)	Zn3^xviii^—Zn4—Ba1^ix^	74.638 (9)
Zn3—Zn2—Cu1^iv^	111.420 (16)	Zn3^viii^—Zn4—Ba1^ix^	76.705 (9)
Zn3^vi^—Zn2—Cu1^iv^	56.601 (10)	Zn3^vii^—Zn4—Ba1^ix^	123.655 (15)
Cu1—Zn2—Cu1^iv^	105.245 (11)	Cu1—Zn4—Ba1^ix^	67.126 (9)
Zn3^x^—Zn2—Cu1^iii^	112.000 (16)	Zn4^xii^—Zn4—Ba1^ix^	165.22 (2)
Zn3^iii^—Zn2—Cu1^iii^	56.189 (10)	Zn4^xiii^—Zn4—Ba1^ix^	63.692 (10)
Zn3—Zn2—Cu1^iii^	56.601 (10)	Ba1^xvii^—Zn4—Ba1^ix^	129.234 (6)
Zn3^vi^—Zn2—Cu1^iii^	111.420 (16)	Zn3^xvii^—Zn4—Ba1^vii^	74.638 (9)
Cu1—Zn2—Cu1^iii^	105.245 (11)	Zn3^xviii^—Zn4—Ba1^vii^	121.676 (15)
Cu1^iv^—Zn2—Cu1^iii^	130.57 (2)	Zn3^viii^—Zn4—Ba1^vii^	123.655 (15)
Zn3^x^—Zn2—Ba1	77.010 (12)	Zn3^vii^—Zn4—Ba1^vii^	76.705 (9)
Zn3^iii^—Zn2—Ba1	77.010 (12)	Cu1—Zn4—Ba1^vii^	67.126 (9)
Zn3—Zn2—Ba1	77.635 (12)	Zn4^xii^—Zn4—Ba1^vii^	63.692 (10)
Zn3^vi^—Zn2—Ba1	77.635 (12)	Zn4^xiii^—Zn4—Ba1^vii^	165.22 (2)
Cu1—Zn2—Ba1	129.470 (17)	Ba1^xvii^—Zn4—Ba1^vii^	129.234 (6)
Cu1^iv^—Zn2—Ba1	65.284 (11)	Ba1^ix^—Zn4—Ba1^vii^	101.532 (12)
Cu1^iii^—Zn2—Ba1	65.284 (11)	Zn3^xvii^—Zn4—Ba1^xv^	63.718 (10)
Zn3^x^—Zn2—Ba1^vii^	121.722 (15)	Zn3^xviii^—Zn4—Ba1^xv^	63.718 (11)
Zn3^iii^—Zn2—Ba1^vii^	74.105 (9)	Zn3^viii^—Zn4—Ba1^xv^	139.660 (11)
Zn3—Zn2—Ba1^vii^	75.842 (9)	Zn3^vii^—Zn4—Ba1^xv^	139.660 (11)
Zn3^vi^—Zn2—Ba1^vii^	122.810 (14)	Cu1—Zn4—Ba1^xv^	96.897 (15)
Cu1—Zn2—Ba1^vii^	67.425 (9)	Zn4^xii^—Zn4—Ba1^xv^	106.854 (15)
Cu1^iv^—Zn2—Ba1^vii^	165.984 (15)	Zn4^xiii^—Zn4—Ba1^xv^	106.854 (15)
Cu1^iii^—Zn2—Ba1^vii^	63.436 (8)	Ba1^xvii^—Zn4—Ba1^xv^	134.919 (13)
Ba1—Zn2—Ba1^vii^	128.704 (6)	Ba1^ix^—Zn4—Ba1^xv^	63.342 (8)
Zn3^x^—Zn2—Ba1^ix^	74.105 (9)	Ba1^vii^—Zn4—Ba1^xv^	63.342 (8)

Symmetry codes: (i) *x*, *y*, *z*+1; (ii) *x*+1/2, *y*, −*z*+1/2; (iii) −*x*+1/2, −*y*, *z*+1/2; (iv) −*x*+1/2, −*y*+1, *z*+1/2; (v) *x*, −*y*+1/2, *z*+1; (vi) *x*, −*y*+1/2, *z*; (vii) −*x*+1/2, −*y*, *z*−1/2; (viii) −*x*+1/2, *y*+1/2, *z*−1/2; (ix) −*x*+1/2, −*y*+1, *z*−1/2; (x) −*x*+1/2, *y*+1/2, *z*+1/2; (xi) *x*, −*y*−1/2, *z*; (xii) −*x*, −*y*, −*z*; (xiii) −*x*, −*y*+1, −*z*; (xiv) *x*, *y*, *z*−1; (xv) *x*−1/2, *y*, −*z*+3/2; (xvi) −*x*+1, −*y*, −*z*+1; (xvii) *x*−1/2, *y*, −*z*+1/2; (xviii) *x*−1/2, −*y*+1/2, −*z*+1/2.
